# Clinical prediction models for serious infections in children: external validation in ambulatory care

**DOI:** 10.1186/s12916-023-02860-4

**Published:** 2023-04-18

**Authors:** David A. G. Bos, Tine De Burghgraeve, An De Sutter, Frank Buntinx, Jan Y. Verbakel

**Affiliations:** 1 EPI-Centre, Department of Public Health and Primary Care, KU Leuven, 7 Kapucijnenvoer block H - Box 7001, Leuven, 3000 Belgium; 2 Department of Family Practice and Primary Health Care, Ghent University, Ghent, Belgium; 3 Department of Public Health and Primary Care, KU Leuven, Leuven, Belgium; 4 Research Institute Caphri, Maastricht University, Maastricht, The Netherlands; 5 NIHR Community Healthcare Medtech and IVD Cooperative, Nuffield Department of Primary Care Health Sciences, University of Oxford, Oxford, UK

**Keywords:** Serious infections, Children, Clinical prediction models, External validation, General practice, Paediatrics, Emergency medicine, Diagnostic accuracy, Model updating

## Abstract

**Background:**

Early distinction between mild and serious infections (SI) is challenging in children in ambulatory care. Clinical prediction models (CPMs), developed to aid physicians in clinical decision-making, require broad external validation before clinical use. We aimed to externally validate four CPMs, developed in emergency departments, in ambulatory care.

**Methods:**

We applied the CPMs in a prospective cohort of acutely ill children presenting to general practices, outpatient paediatric practices or emergency departments in Flanders, Belgium. For two multinomial regression models, Feverkidstool and Craig model, discriminative ability and calibration were assessed, and a model update was performed by re-estimation of coefficients with correction for overfitting. For two risk scores, the SBI score and PAWS, the diagnostic test accuracy was assessed.

**Results:**

A total of 8211 children were included, comprising 498 SI and 276 serious bacterial infections (SBI). Feverkidstool had a C-statistic of 0.80 (95% confidence interval 0.77–0.84) with good calibration for pneumonia and 0.74 (0.70–0.79) with poor calibration for other SBI. The Craig model had a C-statistic of 0.80 (0.77–0.83) for pneumonia, 0.75 (0.70–0.80) for complicated urinary tract infections and 0.63 (0.39–0.88) for bacteraemia, with poor calibration. The model update resulted in improved C-statistics for all outcomes and good overall calibration for Feverkidstool and the Craig model. SBI score and PAWS performed extremely weak with sensitivities of 0.12 (0.09–0.15) and 0.32 (0.28–0.37).

**Conclusions:**

Feverkidstool and the Craig model show good discriminative ability for predicting SBI and a potential for early recognition of SBI, confirming good external validity in a low prevalence setting of SBI. The SBI score and PAWS showed poor diagnostic performance.

**Trial registration:**

ClinicalTrials.gov, NCT02024282. Registered on 31 December 2013.

**Supplementary Information:**

The online version contains supplementary material available at 10.1186/s12916-023-02860-4.

## Background

Examining a feverish child is part of daily practice for general practitioners (GPs), paediatricians and emergency physicians. In most cases, it concerns a mild, often viral infection with a favourable natural course [1–5]. Physicians however must always be cautious of potentially serious infections (SI) requiring more extensive treatment or hospital admission. Late recognition of these conditions may cause serious complications and possibly even death. The distinction between these mild and serious infections is difficult at an early stage [6]. The physician must decide solely based on clinical history taking and examination whether he can rule out SI or if immediate medical treatment or referral to secondary care is needed. Physician’s evaluation shows a low sensitivity, especially when specific signs or symptoms are missing [3]. This diagnostic problem may cause a delay in the appropriate antibiotic treatment or may lead to unnecessary antibiotic prescriptions, aggravating the problem of antibiotic resistance [3, 7].

To aid doctors in clinical decision-making, several clinical prediction models (CPMs) have been developed. CPMs calculate the risk of SI from different variables such as demographic factors, medical history, clinical examination, general clinical impression and physician’s gut feeling something is wrong; in some CPMs, point-of-care (POC) tests are included as well [2–5, 8]. CPMs take several forms. First, we have risk scores where each variable counts for a specified number of points. Following management decisions depend on predefined risk cut-offs. Second is binomial logistic regression models (LRMs) with a binary outcome, calculating the probability of one specific disease and presenting it as a percentage. Third is an extension of these binomial regression models to multinomial LRMs (more than 2 outcome categories) which estimate the probabilities of several diseases simultaneously. This resembles more closely the traditional clinical diagnostic process where multiple diseases are considered in a differential diagnosis. Some CPMs have proven excellent clinical performance compared to physician’s evaluations [3].

Deriving CPMs has an inherent risk of overfitting, where the prediction is fitted too strongly to the original derivation data. Consequently, the model underperforms in populations differing from the original derivation cohort, necessitating the external validation of CPMs in independent populations [9–11].

A previous external validation study of CPMs for acutely ill children in ambulatory care found promising rule-out values, with still a percentage of residual uncertainty [12]. More recently, one CPM has proven extremely sensitive to external validation in ambulatory care in identifying acutely ill children at risk for hospital admission for a serious infection [2]. Although most acutely ill children present in primary care [12], we are not aware of other recent CPMs for serious infections in children in ambulatory care. Therefore, we wished to investigate whether CPMs derived in EDs could be applicable in primary care settings as well. From a recent systematic review on CPMs for feverish children in the emergency department (ED), we identified three clinical prediction models potentially applicable in primary care: two multinomial LRMs and one risk score, based on a binomial LRM [3–5, 13]. For these three models, external validation has been performed in EDs, but not yet in broader ambulatory care [4, 13–17]. We searched the references of the included studies for other relevant CPMs, and we identified one additional CPM, another risk score, assessing the broader risk of serious illness [8]. In our study, we aimed to externally validate and, if applicable, update these four CPMs for SI in children in a population of children with an acute illness presenting to ambulatory care in Belgium.

## Methods

We performed this secondary analysis of prospectively gathered observational data to externally validate these four CPMs for SI in children by assessing discriminative value and calibration. Subsequently, we performed a model update of the LRMs in our dataset. The study is reported in agreement with the TRIPOD guideline on transparent reporting of multivariable prediction models for diagnosis [18].

### External validation dataset

From 15 February 2013 to 28 February 2014, children from 1 month to 16 years with an acute illness were included in 92 GP practices, 6 ambulatory paediatric practices and 6 EDs in Flanders, Belgium, as part of the ERNIE2 study [2]. Further details on the inclusion and exclusion criteria are reported elsewhere [19]. Seventy-four diagnostic items were registered. Age is reported as mean and interquartile range.

In the ERNIE2 study, SI was defined as an infection requiring hospital admission for more than 24 h. It comprised both the serious bacterial infections sepsis and bacteraemia, meningitis, pneumonia, osteomyelitis, cellulitis and cUTI as well as appendicitis, gastro-enteritis with dehydration and viral respiratory tract infection with hypoxia [19]. The diagnosis was checked in the GP’s electronic medical records and the hospital records. Depending on the condition, microbiological, biochemical, histological, radiological or clinical criteria were required for a definite diagnosis [19]. An adjudication committee of clinicians with expertise in acute paediatric care assessed all available information of cases with no definite diagnosis in the medical record or after the interview with the GP and assigned outcome by consensus [2]. Since the distinction between viral and bacterial gastro-enteritis could not be made in the ERNIE2 dataset, the data of participants with gastro-enteritis were excluded from the analysis of SBI in the current study.

### Clinical prediction models

The first CPM, Feverkidstool, is a multinomial LRM predicting the risk of pneumonia and other serious bacterial infections (SBI) in feverish children from 10 clinical variables and a POC CRP test [4]. In the derivation of the Feverkidstool, positive cultures of normal sterile sites or consensus diagnosis were required [4]. In the main recruiting hospital, the diagnosis of pneumonia was based on radiological criteria, assessed by two radiologists, blinded to the clinical information; in the other recruiting hospitals, assessment of chest radiographies was performed by a single radiologist, not blinded to the clinical information.

The second model, a CPM constructed by Craig et al. (hereafter named the Craig model), is a multinomial LRM predicting the risk of complicated urinary tract infections (cUTI), pneumonia and bacteraemia in feverish children [3]. The model consists of 26 items, including several variables from history taking. Craig et al. divided the diagnoses of UTI, pneumonia and bacteraemia into definite and probable, based on microbiological and radiological criteria, which were nearly identical to our diagnostic criteria [3]. All probable cases were reviewed by a final diagnosis committee, composed of two specialist paediatricians and a radiologist for pneumonia, blinded to the clinical information. The presence or absence of bacterial infections was based on consensus.

In our validation dataset, the broader inclusion criterion of acutely ill children presenting at ambulatory care was used, whereas the derivation of Feverkidstool and the Craig model inclusions were limited to children presenting with fever at the ED [3, 4]. Feverkidstool and the Craig model were developed to predict the risk of SBI, excluding viral infections. Unlike in the ERNIE2 cohort, the Feverkidstool and Craig model studies did not require hospital admission as a marker of SBI. Children with SBI receiving outpatient care therefore would have been included in these studies but were not considered SBI in our cohort.

The third CPM, the SBI score, is a risk score assessing the risk of SBI (excluding viral infections) in acutely ill children. It consists of eight clinical items and was derived from a binomial LRM (hereafter named the SBI model) [5]. As in the ERNIE2 cohort, the SBI score study required hospital admission as a marker of SBI, plus disease-specific findings such as positive cultures in sterile sites or radiological signs, nearly identical to our diagnostic criteria.

The fourth CPM, the Pediatric Advanced Warning Score (PAWS), is a risk score based on seven age-specific vital parameters in children [8]. PAWS was developed to predict the risk of serious illness, broader than serious infections alone, in the ED. In a pilot case–control study, admission from the ED to the paediatric intensive care unit was used as a marker of serious illness, compared to children admitted to the general paediatric ward. We applied PAWS to our clinical endpoint of serious infections requiring hospital admission (including viral infections). See Additional file 1: Tables S1-S7 for more details on participants, predictor and outcome variables and regression coefficients in the derivation studies [20–26].

### Model validation

The presence of each predictor variable from the different models was evaluated in the ERNIE2 database. If variables were similar, but not exactly corresponding, a proxy was used. Missing values were considered non-deviant (see the ‘Discussion’ section), except for participants with missing data on outcome, age, sex or temperature, which were excluded. An outcome value was required to correctly perform the analyses, age was required for age-specific vital signs, temperature was registered twice in our dataset (both recorded by the parents and recorded by the physician) and was required as an absolute value in the algorithm of Feverkidstool, and for sex, no non-deviant result could be imputed.

To assess the discriminative ability of the multinomial LRMs, Feverkidstool and Craig model, we calculated the conditional pairwise concordance statistic, hereafter referred to as the C-statistic, using the original reported coefficients and intercepts. The conditional risk was calculated by dividing the probability of the disease of interest by the sum of the probabilities of the disease of interest and of the absence of SBI. The C-statistic and its 95% confidence interval (CI) were calculated as the area under the receiver operating characteristic curve (AUC) of this conditional risk with the R statistical software version 4.1.2 (R Foundation for Statistical Computing, Vienna, Austria) [27] using the pROC-package [28]. To assess the calibration of the models, we constructed multinomial calibration plots and calculated calibration intercepts and calibration slopes using the non-parametric framework by Van Hoorde et al. [24, 25] (see Additional file 1 for more details on calibration intercepts and slopes).

For the binomial SBI model, discrimination and calibration were evaluated using the val.prob.ci.2 function, an adaptation of the rms package [21].

For the risk scores SBI score and PAWS, sensitivity, specificity, positive (LR( +)) and negative likelihood ratio (LR( −)) and the respective 95%-CIs were calculated at the cut-offs proposed by the original authors.

### Model update

First, we performed logistic recalibration of the LRMs [24, 25]. Next, we performed model revision by refitting the variables in our dataset and re-estimating individual coefficients. Finally, we applied uniform shrinkage of the revised coefficients towards the recalibrated values using a heuristic shrinkage factor to correct for overfitting on our dataset [22]. For refitting of the multinomial LRMs Feverkidstool and Craig model, the multinom-function of the nnet-package was used [26], and for the binomial LRM SBI model, the rms-package was used [23] (see Additional file 1 for more details on logistic recalibration and the heuristic shrinkage factor).

We assessed discrimination and calibration of the updated models as described above and calculated sensitivity, specificity and positive and negative likelihood ratio at low- and high-risk cut-offs.

## Results

### Participants

A total of 8962 acutely ill children were included, of which 730 were excluded due to missing essential data (age, sex, temperature, outcome) and 21 for exceeding the age range, leading to 8211 participants in the current analysis. SI was established in 498 children, leading to an intermediate prevalence of 6.1% (5.6–6.6%) in our combined ambulatory setting [12]. SBI was diagnosed in 276 children, resulting in a prevalence of 3.4% (3.0–3.8%). These SI most often affected the youngest children. Two-thirds of SI consisted of pneumonia and gastro-enteritis with dehydration. The participant characteristics are summarized in Table 1 (see Additional file 1: Tables S1, S3, S5 and S7 for a comparison between the validation and the derivation cohorts).

**Table 1:** Baseline characteristics of the included children

	Children with serious infection (*n* = 498)	Children without serious infection (*n* = 7713)
Median age in years (interquartile range)	1.62 (0.78–3.79)	1.97 (0.99–4.02)
Sex, male (%)	268 (54%)	4133 (54%)
Inclusion by general practitioner (*n* = 2902)	23	2879
Inclusion by ambulatory paediatrician (*n* = 2719)	109	2610
Inclusion by emergency department (*n* = 2590)	366	2224
Outcome (hospital admission > 24 h with)		
Abscess/cellulitis	10	0
Appendicitis	15	0
Complicated urinary tract infection	57	0
Gastro-enteritis with dehydration (bacterial and viral)	162	0
Meningitis (bacterial and viral)	16	0
Osteomyelitis	0	0
Pneumonia	171	0
Sepsis/bacteraemia	7	0
Viral respiratory tract infection with hypoxia	60	0
Non-serious infection	0	7713

### Model validation

For the Feverkidstool, all 11 variables were available in the ERNIE2 database. For the Craig model, 11 of 26 variables exactly matched the available variables. The variables ‘rash’, ‘stridor’ and ‘audible wheeze’ were not systematically registered in our database but had been registered by physicians in the free text space ‘other signs of illness’. For nine variables, a proxy was used of which eight proxies closely resembled the original variables. For urinary symptoms, only the weak proxy ‘does your child urinate less’ was available. The categories ‘felt hot’ and meningococcal and pneumococcal vaccination were not available. Four of the eight variables of the SBI model and SBI score matched the variables in the ERNIE2 database. For the other four variables, a proxy was used with a close resemblance to the original variable. For PAWS, five of the seven variables were available. For the missing variables ‘work of breathing’ and the AVPU scale, two moderate-quality proxies were used (see Additional file 1: Tables S1, S3, S5 and S7 for a detailed description of the proxies).

Feverkidstool and the Craig model showed good discriminative values with C-statistics of 0.80 (0.77–0.84) for pneumonia and 0.74 (0.70–0.79) for other SBI by Feverkidstool, and 0.80 (0.77–0.83) for pneumonia and 0.75 (0.70–0.80) for cUTI by the Craig model. For the SBI model and the prediction of bacteraemia by the Craig model, we found poor discriminative ability (C-statistics of 0.66 (0.59–0.73) and 0.63 (0.39–0.88), respectively) (Table 2 (A)).

**Table 2:** Diagnostic performance of the clinical prediction models

**A: C-statistics of the logistic regression models**				
		Original model (95%-CI)	Updated model (95%-CI)	
**Feverkidstool** (*n* = 8049)	*Pneumonia*	0.80 (0.77–0.84)	0.83 (0.80–0.86)	
	*Other SBI*	0.74 (0.70–0.79)	0.78 (0.74–0.83)	
**Craig model** (*n* = 8211)	*Pneumonia*	0.80 (0.77–0.83)	0.83 (0.80–0.86)	
	*Complicated UTI*	0.75 (0.70–0.80)	0.86 (0.83–0.90)	
	*Bacteraemia*	0.63 (0.39–0.88)	0.80 (0.66–0.94)	
**SBI-model** (*n* = 8049)		0.66 (0.59–0.73)	0.67 (0.60–0.73)	
**B: Calibration intercepts and calibration slopes of the logistic regression models**				
	Original model		Updated model	
	Calibration intercept (95%-CI)	Calibration slope (95%-CI)	Calibration intercept (95%-CI)	Calibration slope (95%-CI)
**Feverkidstool**				
*Pneumonia vs. absence of SBI*	0.09 (− 0.07 to 0.24)	1.01 (0.87 to 1.14)	0.00 (− 0.16 to 0.16)	1.04 (0.92 to 1.17)
*Other SBI vs. absence of SBI*	− 2.76 (− 2.96 to − 2.56)	0.50 (0.38 to 61)	0.00 (− 0.20 to 0.20)	1.05 (0.86 to 1.25)
**Craig model**				
*Pneumonia vs. absence of SBI*	− 0.87 (− 1.03 to − 0.71)	0.72 (0.63 to 0.81)	0.00 (− 0.16 to 0.16)	1.05 (0.93 to 1.18)
*Complicated UTI vs. absence of SBI*	− 1.33 (− 1.59 to − 1.06)	0.69 (0.49 to 0.88)	0.00 (− 0.26 to 0.26)	1.27 (1.00 to 1.55)
*Bacteraemia vs. absence of SBI*	− 1.31 (− 2.06 to − 0.55)	0.30 (− 0.10 to 0.71)	0.00 (− 0.75 to 0.75)	0.34 (0.02 to 0.65)
**SBI model**	− 4.97 (− 5.10 to − 4.85)	0.71 (0.60 to 0.83)	0.00 (− 0.12 to 0.12)	1.03 (0.87 to 1.20)
**C: Diagnostic test parameters for updated regression models and original risk scores**				
	Sensitivity (95%-CI)	Specificity (95%-CI)	LR( +) (95%-CI)	LR( −) (95%-CI)
**Updated Feverkidstool**				
*Pneumonia*				
*Risk* ≥ *2.5%*	0.71 (0.64–0.78)	0.77 (0.76–0.78)	3.09 (2.79–3.42)	0.37 (0.29–0.47)
*Risk* ≥ *10%*	0.29 (0.22–0.36)	0.98 (0.97–0.98)	12.40 (9.41–16.36)	0.73 (0.66–0.80)
*Risk* ≥ *30%*	0.08 (0.04–0.13)	1.00 (1.00–1.00)	31.52 (15.83–62.78)	0.93 (0.89–0.97)
*Other SBI*				
*Risk* ≥ *2.5%*	0.51 (0.41–0.61)	0.87 (0.86–0.88)	3.91 (3.21–4.75)	0.56 (0.46–0.68)
*Risk* ≥ *10%*	0.04 (0.01–0.10)	1.00 (0.99–1.00)	10.29 (3.69–28.67)	0.96 (0.93–1.00)
*Risk* ≥ *30%*	0 (0–0.04)	1 (1–1)	NA	1 (1–1)
**Updated Craig model**				
*Pneumonia*				
*Risk* ≥ *2.5%*	0.69 (0.61–0.76)	0.81 (0.80–0.81)	3.56 (3.19–3.97)	0.38 (0.31–0.48)
*Risk* ≥ *10%*	0.27 (0.21–0.35)	0.97 (0.97–0.98)	10.89 (8.24–14.39)	0.74 (0.68–0.82)
*Risk* ≥ *30%*	0.08 (0.04–0.13)	1.00 (1.00–1.00)	43.66 (20.84–91.47)	0.93 (0.89–0.97)
*Complicated UTI*				
*Risk* ≥ *2.5%*	0.23 (0.13–0.36)	0.96 (0.95–0.96)	5.62 (3.45–9.16)	0.80 (0.70–0.93)
*Risk* ≥ *10%*	0.04 (0.00–0.12)	1.00 (1.00–1.00)	40.87 (8.68–192.51)	0.97 (0.92–1.01)
*Risk* ≥ *30%*	0 (0–0.06)	1 (1–1)	NA	1 (1–1.00)
*Bacteraemia*				
*Risk* ≥ *2.5%*	0 (0–0.41)	1.00 (1.00–1.00)	0 (0–0.13)	1.00 (1.00–1.00)
*Risk* ≥ *10%*	0 (0–0.41)	1.00 (1.00–1.00)	0 (0–0.97)	1.00 (1.00–1.00)
*Risk* ≥ *30%*	0 (0–0)	1 (1–1)	NA	1 (1–1)
**Updated SBI model**				
*Risk* ≥ *2.5%*	0.93 (0.89–0.95)	0.25 (0.24–0.26)	1.23 (1.19–1.27)	0.30 (0.19–0.45)
*Risk* ≥ *10%*	0.11 (0.07–0.15)	0.98 (0.98–0.99)	6.23 (4.25–9.15)	0.91 (0.87–0.95)
*Risk* ≥ *30%*	0.01 (0.00–0.03)	1.00 (1.00–1.00)	3.78 (0.87–16.46)	0.99 (0.98–1.00)
**SBI score**				
*Score* ≤ *5*	0.11 (0.08–0.15)	0.98 (0.98–0.98)	5.81 (4.15–8.15)	0.90 (0.87–0.94)
*Score* > *8*	0.01 (0.00–0.02)	1.00 (1.00–1.00)	4.20 (0.93–18.87)	0.99 (0.98–1.00)
**PAWS**				
*Score* ≥ *3*	0.32 (0.28–0.37)	0.86 (0.85–0.87)	2.28 (1.98–2.61)	0.79 (0.74–0.84)

2.5% was chosen as a low-risk cut-off, and 10% and 30% were chosen as high-risk cut-offs. Diagnostic test parameters were calculated at the proposed risk cut-offs for PAWS and the SBI score, with a score ≤ 5 as a low-risk cut-off and > 8 as a high-risk cut-off for the SBI score

*SBI* Serious bacterial infections, *UTI* Urinary tract infection, *CI* Confidence interval, *LR(* +*)* Positive likelihood ratio, *LR( −)* Negative likelihood ratio

**Fig. 1:**
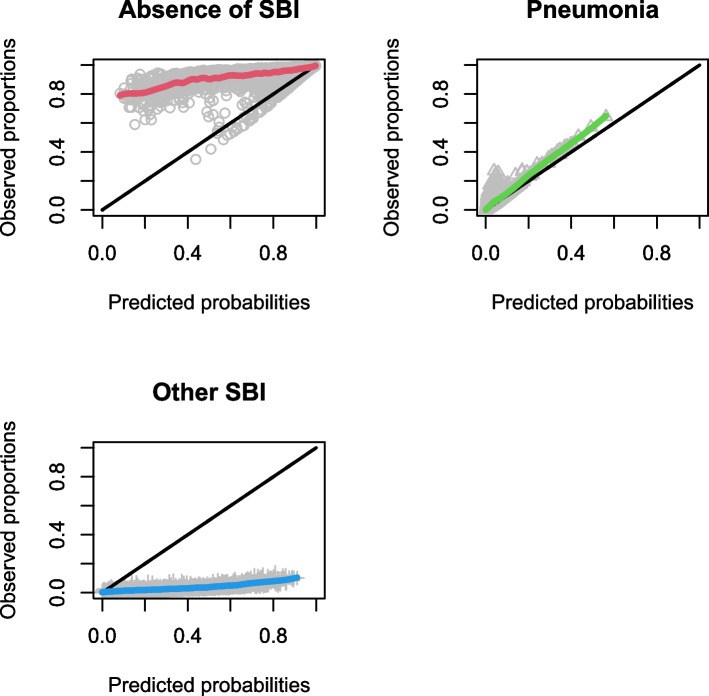
Non-parametric multinomial calibration plots of Feverkidstool. SBI, serious bacterial infections

**Fig. 2:**
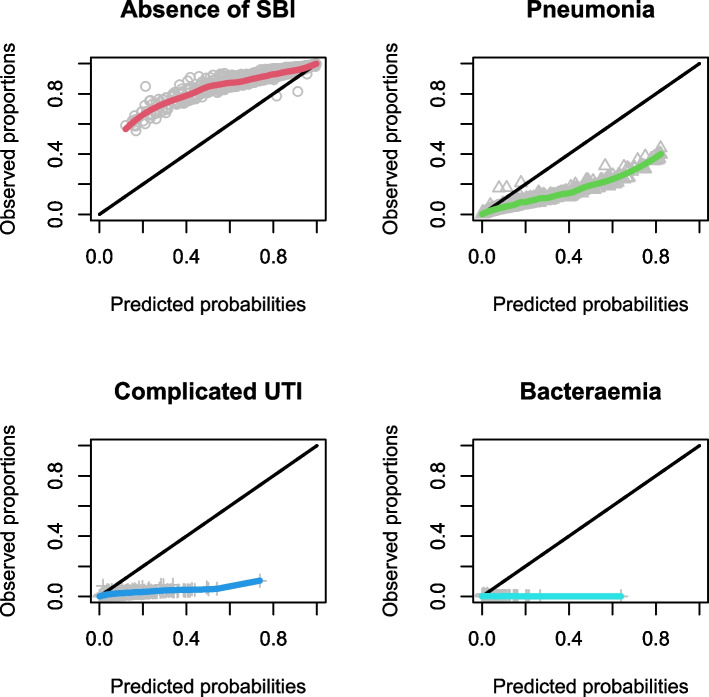
Non-parametric multinomial calibration plots of the Craig model. SBI, serious bacterial infections; UTI, urinary tract infections

**Fig. 3:**
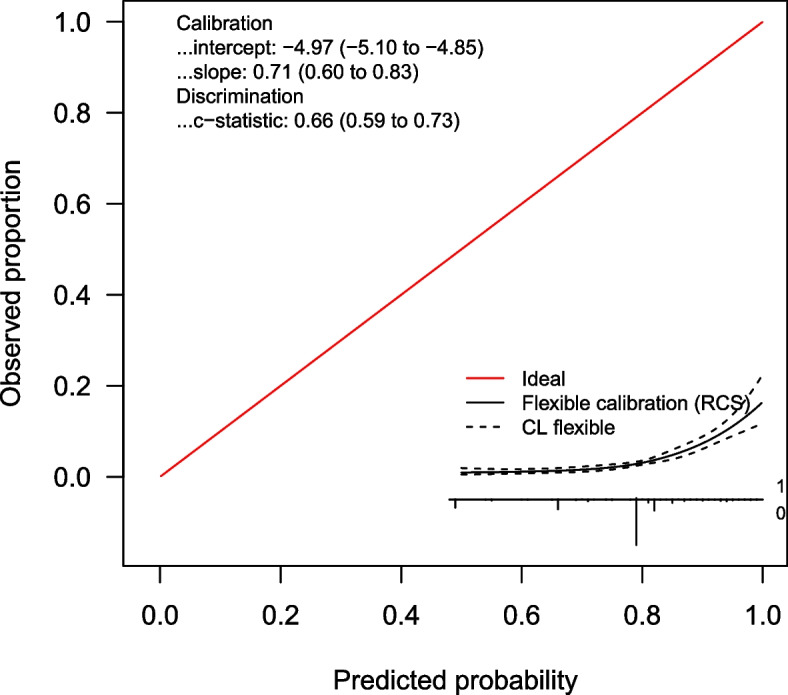
Calibration curve of the SBI model. SBI, serious bacterial infections; RCS, restricted cubic splines; CL, confidence limit

Calibration plots for the LRMs are shown in Figs. 1, 2 and 3, and calibration intercepts and slopes are summarized in Table 2 (B). Feverkidstool showed a mild underestimation of the probability of pneumonia and a large overestimation of the risk of other SBI, resulting in a strong underestimation of the absence of SBI. The Craig model overestimated the risk for all outcome categories and thereby underestimated the absence of SBI. Risk predictions for SBI by the SBI model were gravely overestimated.

The analytical performance of the SBI score and PAWS are summarized in Table 2 (C). Both models showed extremely low sensitivities (< 32%).

Sensitivity analyses for the original age ranges and participants included in the emergency department are available in Additional file 1: Tables S8-S11.

### Model update

**Fig. 4:**
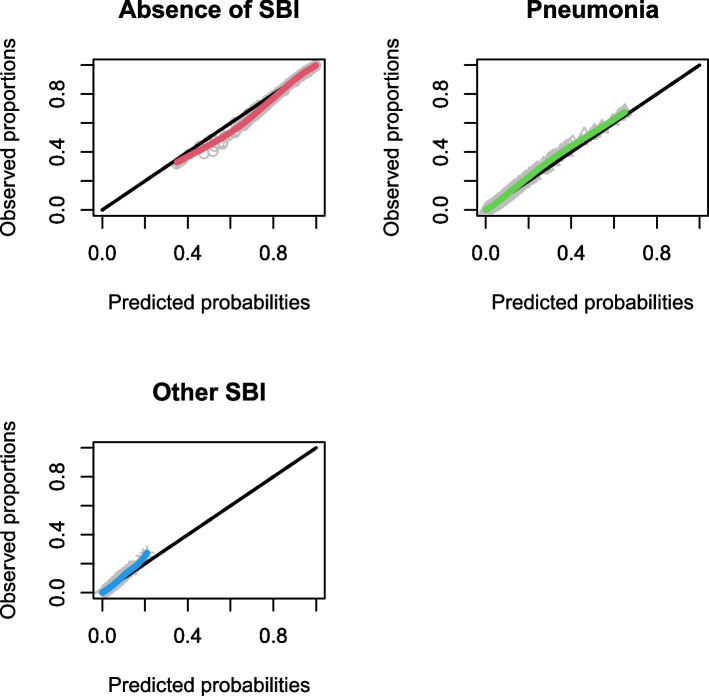
Non-parametric multinomial calibration plots of updated Feverkidstool. SBI, serious bacterial infections

**Fig. 5:**
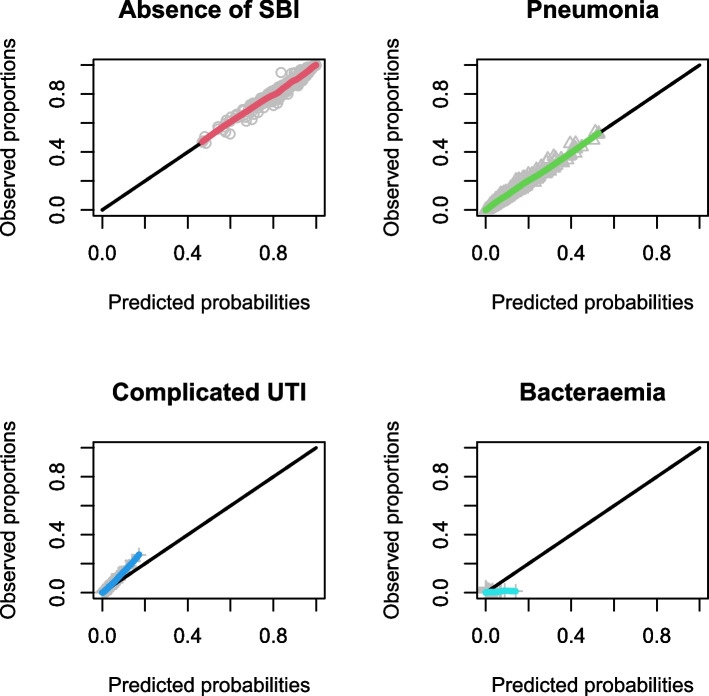
Non-parametric multinomial calibration plots of the updated Craig model. SBI, serious bacterial infections; UTI, urinary tract infections

**Fig. 6:**
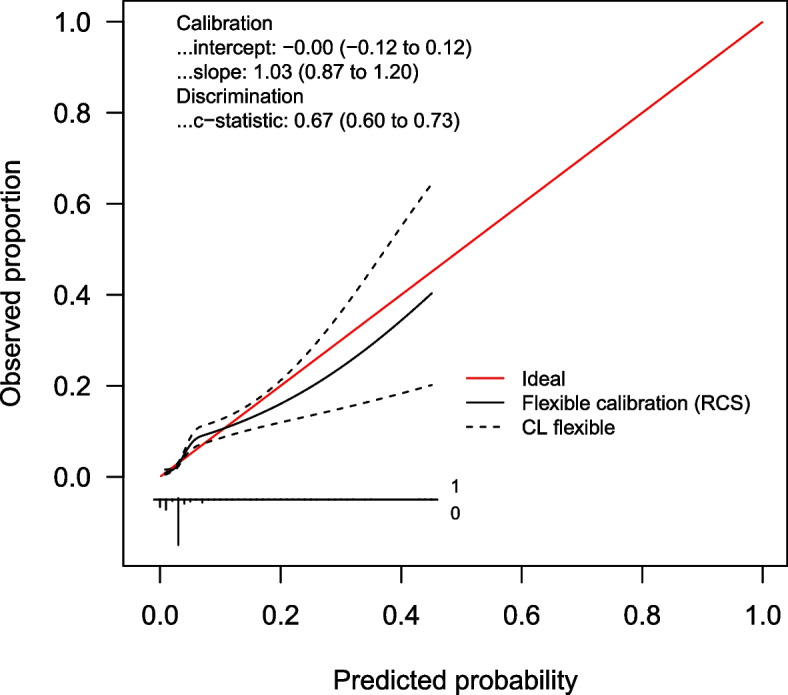
Calibration curve of updated SBI model. SBI, serious bacterial infections; RCS, restricted cubic splines; CL, confidence limit

The discriminative ability increased for both Feverkidstool and the Craig model, with the strongest increases for cUTI and bacteraemia (C-statistics of 0.86 (0.83–0.86) and 0.80 (0.66–0.94), respectively). The SBI model barely improved after updating (Table 2 (A)). Calibration of all models improved markedly with accurate risk predictions for pneumonia and absence of SBI by both Feverkidstool and the Craig model. Predictions for bacteraemia remained overestimated, and for cUTI, we found mild underestimation of risk. Updated regression coefficients are available in Additional file 1: Tables S2, S4 and S6. Calibration plots of the updated models are shown in Figs. 4, 5 and 6, and calibration intercepts and slopes of the updated models are summarized in Table 2 (B).

Sensitivity was low for all outcome categories of the updated Feverkidstool and Craig model (< 71%). Especially for bacteraemia, the Craig model failed to correctly identify any of the cases. Specificities on the other hand were very high for all outcome categories from risk cut-offs of 10%. The sensitivity of the updated SBI model however was good at a low-risk cut-off of 2.5% risk, at the cost of a very low specificity (Table 2 (C)).

## Discussion

### Main findings

In this study, we performed an external validation of four CPMs for SI in 8211 children from 1 month to 16 years presenting with acute illness in ambulatory care. The multinomial LRMs Feverkidstool and Craig model showed varying C-statistics for the different outcomes ranging from 0.63 to 0.80. Predictions of pneumonia by Feverkidstool were well calibrated, but the other outcomes showed weak calibration. After the model update, Feverkidstool and the Craig model achieved good discriminative ability with C-statistics for the different outcomes ranging from 0.78 to 0.86 and good overall calibration. At low-risk cut-offs, however, sensitivities ranged from 0 to 0.71, and negative likelihood ratios ranged from 1 to 0.37, limiting the rule-out value for SBI. Specificity (> 0.97) and positive likelihood ratios (> 10.29) on the other hand were very high at higher-risk cut-offs. The models therefore seem more suited for ruling in than for ruling out SI in ambulatory care. The risk scores SBI score and PAWS performed very poorly in our cohort with extremely low sensitivities of 0.11 and 0.32, respectively. They appear unfit to effectively rule out SI in the ambulatory setting.

### Strengths and limitations

This validation was performed in a very large, prospective cohort in a clinically relevant, broad ambulatory setting. It is the first validation study of these models to include GP and paediatric outpatient practices, representing a low-prevalence setting for SBI. However, the vast majority of SI were diagnosed in the ED, making separate analyses for those settings unreliable.

The CPMs were developed in different countries with distinct healthcare systems, vaccination policy and vaccination uptake. Further heterogeneity is introduced by the different variables and the differences between the derivation and validation cohorts. For the Feverkidstool, the most accurate external validation could be performed, since all variables were present in the validation dataset and the derivation and validation cohort had similar age and sex distributions. The proportion of SBI was clearly higher (12% vs. 3%), reflecting the difference in setting. The Craig model had the lowest proportion of variables available, yet still showed good diagnostic performance. It was derived in younger children from 0 to 5 years, while we applied it to children from 1 month to 16 years. For the SBI score, four good-quality proxies were used on a total of eight variables. Children in the derivation cohort were on average slightly younger (median 1.58 years vs. 1.96 years), and had a similar proportion of SBI (3.8% vs. 3.4%). For PAWS two moderate-quality proxies were available on seven variables, mildly reducing the accuracy of our external validation.

Inevitably, as in any large study in daily clinical practice, not all data were registered completely. We performed single imputation of the missing values and considered them non-deviant, from the assumption that normal parameters are less frequently registered in a child in good clinical condition with a low probability of SI, especially by GPs. Further research focussing on developing more appropriate methodology to perform multiple imputation for multinomial models could facilitate future analyses for similar research questions [29].

### Comparison with existing literature

The Feverkidstool is by far the most studied model, with several external validation studies in EDs both by the original research team and independent external validations [4, 13–17, 30]. We found a comparable C-statistic for pneumonia as in the original derivation study, but a clearly lower C-statistic for other SBI [4]. In external validation studies, C-statistics ranged from 0.72 to 0.89 for pneumonia and from 0.68 to 0.82 for other SBI, increasing after the model update [13–17]. Sensitivities at low-risk cut-offs were clearly higher compared to our findings [4, 16].

Decision rules in the ED based on the Feverkidstool did not report a substantial impact on the patient outcome or reduction of overall antibiotic prescription [17, 31]. The decision rule however proved non-inferior to usual care and resulted in fewer antibiotic prescriptions in children with low to intermediate risk for SBI, suggesting more appropriate antibiotic prescriptions [31], and was cost-saving by reducing hospitalization and parental absenteeism [32].

The discriminative value of the Craig model was better in the original study [3] and lower in a validation study in children under three months [13]. The AUC of the original SBI model was 0.77 [5], but similar to our findings, this could not be confirmed at external validation [13]. The developers of PAWS found a sensitivity of 70% and a specificity of 90%, contrasting strongly with our findings [8].

### Impact on research

Our study contributes to the broad external validation required before using a CPM reliably in daily practice [11]. Children with acute illnesses most often consult GP practices, yet less studies are conducted in general practice [12], necessitating the need to validate existing prediction rules in this setting.

In our study, we found a comparable performance between the Craig model with a large number of clinical variables on three outcome categories [3] and Feverkidstool with less variables, but including a POC CRP test and a broad outcome category of other SBI [4]. Both strategies can lead to well-performing models, raising the question whether CPMs for SI in primary care should include both a sufficient number of clinical variables and an additional POC test. Calibration of original models proved best for the Feverkidstool predicting pneumonia. Adding the POC CRP test may therefore prove the model more applicable across settings with less need for more complicated model updating strategies.

### Impact on clinical practice

Implementation of these models may be impacted by the availability of resources. Feverkidstool includes the result of a POC CRP test, which may not be readily available at all sites. Pulse oximetry and measurement of other vital signs in children, useful for Feverkidstool, the SBI score and especially the physiology-based risk score PAWS, are often not routinely available in primary care. The other variables are easy to determine after proper history taking and clinical examination and applicable across different settings and healthcare systems. The Craig model, consisting of 26 clinical signs and symptoms, only requires a thermometer, a stethoscope and an otoscope. The LRMs Feverkidstool, Craig model and SBI model require a simple software application.

The Feverkidstool and the Craig model can support clinical reasoning and the decision-making process of physicians. Combining the numerous findings from history taking and clinical examination at various stages of disease is challenging and may lead to underestimation of SI by discarding information [3] or lead to overestimation of SI from fear to miss serious, but treatable conditions [33]. Physicians appear most successful in correctly identifying serious bacterial illness in the presence of very specific signs and symptoms [3]. A CPM may perform better in identifying SI by combining individual findings in the absence of very specific signs [3, 4]. Rule-out values however seemed limited in our study.

For primary care, these models could be calibrated to be more accurate at lower risk thresholds, making them more suitable for the exclusion of SBI at the cost of more false-positive results and less accurate predictions in higher probability ranges [3]. These models could then be translated into decision rules with clinical management suggestions at predefined risk cut-offs and integrated in electronic health records to aid physicians in real time. Broad impact studies in ambulatory care could further investigate the potential for better recognition of SBI, more appropriate antibiotic prescription and possible cost reduction by applying these decision rules.

## Conclusions

The Feverkidstool and the Craig model show good discrimination for predicting SBI, confirming good external validity in a low prevalence setting of SBI. Their rule-out values at low-risk probabilities were rather limited. Their potential for early recognition and management of SBI should be evaluated in broad-impact studies in ambulatory care. The SBI score and PAWS showed poor performance in our cohort.

## Supplementary Information

**Additional file 1: Table S1-S7.** Comparison of population characteristics, proxy variables, missing data and original and updated coefficients for each model. **Table S8-S11.** Sensitivity analyses for each model.

## Data Availability

Data used during the current study are available from the corresponding author upon reasonable request.

## Abbreviations

AUC: Area under the curve
CI: Confidence interval
CPM: Clinical prediction model
cUTI: Complicated urinary tract infections
ED: Emergency department
GP: General practitioner
LR( −): Negative likelihood ratio
LR( +): Positive likelihood ratio
LRM: Logistic regression model
POC: Point-of-care
SBI: Serious bacterial infections
SI: Serious infections
