# Supplementary material for: Clinical prediction models for serious infections in children: external validation in ambulatory care
Source: BMC Med. 2023 Apr 18;21:151. doi: 10.1186/s12916-023-02860-4 (PMC10114467; doi:10.1186/s12916-023-02860-4)
Supplement: Supplementary file 1 — Additional file 1: Table S1-S7. Comparison of population characteristics, proxy variables, missing data and original and updated coefficients for each model. Table S8-S11. Sensitivity analyses for each model. [file 12916_2023_2860_MOESM1_ESM.pdf]

Supplementary material

1) Calibration and model updating

1) Calibration intercept, calibration slope and logistic recalibration

For the binomial SBI-model calibration and logistic recalibration are assessed as follows:

$$LP_2 = \alpha + \beta * LP$$

with LP_2 the linear predictor of the recalibrated model, α the intercept, β the calibration slope and LP the linear predictor of the original model.[20]

The calibration intercept and calibration slope of the binomial SBI-model were estimated using the val.prob.ci.2-function, an adaptation of the rms-package.[21] To perform logistic recalibration of the binomial LRM SBI-model the intercept α was updated and the calibration slope β was used as a single overall adjustment factor to update the regression coefficients. The intercept and calibration slope were updated by fitting a LRM in the validation sample with the original linear predictor LP as the only covariable using the rms-package.[20,22,23]

For the multinomial feverkidstool with three outcome categories pneumonia, other SBI and absence of SBI, calibration intercepts, calibration slopes and logistic recalibration were assessed as follows:

$$\begin{cases} LP_{2,Pn} = \alpha_1 + \beta_1 * LP_{Pn} + \gamma_1 * LP_{SBI} \\ LP_{2,SBI} = \alpha_2 + \gamma_2 * LP_{Pn} + \beta_2 * LP_{SBI} \end{cases}$$

with $LP_{2,Pn}$ the recalibrated linear predictor for pneumonia and $LP_{2,SBI}$ the recalibrated linear predictor for other SBI with absence of SBI as reference category, α the intercepts, β the coefficients for the corresponding linear predictors and γ the coefficients for the non-corresponding linear predictors, LP_{Pn} the original linear predictor for pneumonia and LP_{SBI} the original linear predictor for other SBI with absence of SBI as reference category.[24,25]

For the multinomial Craig-model with four outcome categories pneumonia, complicated UTI, bacteraemia and absence of SBI, calibration intercepts, calibration slopes and logistic recalibration were assessed as follows:

$$\begin{cases} LP_{2,Pn} = \alpha_1 + \beta_1 * LP_{Pn} + \gamma_1 * LP_{UTI} + \delta_1 * LP_{Ba} \\ LP_{2,UTI} = \alpha_2 + \gamma_2 * LP_{Pn} + \beta_2 * LP_{UTI} + \delta_2 * LP_{Ba} \\ LP_{2,Ba} = \alpha_3 + \gamma_3 * LP_{Pn} + \delta_3 * LP_{UTI} + \beta_3 * LP_{Ba} \end{cases}$$

with $LP_{2,Pn}$ the recalibrated linear predictor for pneumonia, $LP_{2,UTI}$ the recalibrated linear predictor for complicated UTI and $LP_{2,Ba}$ the recalibrated linear predictor for bacteraemia and absence of SBI as reference category, α the intercepts, β the coefficients for the corresponding linear predictors and γ and δ the coefficients for the non-corresponding linear predictors, LP_{Pn} the original linear predictor for pneumonia, LP_{UTI} the original linear predictor for complicated UTI and LP_{Ba} the original linear predictor for bacteraemia with absence of SBI as reference category.

In the estimation of calibration intercepts and slopes, the non-corresponding linear predictors can be considered nuisance parameters, as they do not involve the category of interest.[24] The calibration intercepts were estimated by fixing the coefficients for the non-corresponding linear predictors at 0 and the coefficients of the corresponding linear predictors at 1 and fitting a LRM in the validation sample. The calibration slopes were estimated by fixing the coefficients of the non-corresponding

linear predictors at 0 and fitting a LRM in the validation sample with the original corresponding linear predictors as covariables.
To perform logistic recalibration of the multinomial LRMs feverkidstool and Craig-model the intercept α was updated and coefficients for the corresponding (β) and non-corresponding (γ, δ) linear predictors were estimated to update the regression coefficients. A LRM was fitted in the validation sample with the original linear predictors as the only covariables using the multinom-function of the nnet-package.[24-26]

2) Heuristic shrinkage factor

The heuristic shrinkage factor was calculated as (model χ^2 - df) / model χ^2, where model χ^2 is the difference in -2 log-likelihood between the revised model with re-estimated coefficients and the recalibrated model and df is the difference in degrees of freedom of the two models.[22] The shrunken coefficients were obtained by multiplying the difference in regression coefficients of the re-estimated model and the recalibrated model with the shrinkage factor, thereby pulling the obtained coefficients towards the recalibrated values.[22] Finally the intercept of the shrunken model was re-estimated by fitting a LRM with the linear predictor of the shrunken model as an offset, to allow the sum of predicted probabilities to be equal to the sum of the observed outcomes.[22]

For shrinkage towards recalibrated values with shrinkage factor s:

$$s = \frac{(model\ \chi^2 - df)}{model\ \chi^2}$$

With model χ^2 = -2log-likelihood(recalibrated model) – (-2log-likelihood(revised model))
and df = degrees of freedom(revised model) – degrees of freedom(recalibrated model)

Weighted sum of revised and recalibrated model to obtain revised coefficients with shrinkage towards recalibrated values:

$$shrunken\ coefficients = s * revised\ coefficients + (1 - s) * recalibrated\ coefficients$$

2) Model description, proxy variables used, comparison of population characteristics and missing data

1) Feverkidstool

Table S1: Variables of feverkidstool			
**Variable**	Distribution in derivation study (n=2703)	Distribution in validation study (n=8049)	Missing
Predictors			
**Age** (years) (mean and IQR)	1.77 (0.85 – 3.77)	1.96 (0.97 – 4.01)	0 (0%)
**Gender** (female)	1213 (45%)	3734 (46%)	0 (0%)
**Duration of fever** (days) (median and IQR)	2 (1 – 4)	2 (1 - 3)	1664 (21%)
**Temperature** (°C) (median and IQR)	38.8 (38.0 – 39.5)	39.0 (38.4 – 39.7)	0 (0%)
**Respiratory rate** (breaths per minute) (median and IQR)	32 (24 – 40)	28 (20 – 38)	5719 (71%)
**Heart rate** (beats per minute) (median and IQR)	140 (120 – 160)	110 (91 – 130)	4999 (62%)
**Oxygen saturation <94% 0_2**	80 (3%)	151 (6%)	5412 (67%)
**Capillary refill time ≥3s**	274 (10%)	330 (9%)	4500 (56%)
**Presence of chest wall retractions**	297 (11%)	355 (5%)	221 (3%)
**Ill appearance**	901 (34%)	670 (9%)	275 (3%)
**C-reactive protein** (mg/L) (median and IQR)	15 (8 – 39)	11 (<5 – 30)	1622 (20%)
Outcome			
**SBI**	333 (12%)	274 (3%)	0 (0%)
**Pneumonia**	147 (5%)	171 (2%)	0 (0%)
**Other SBI**	190 (7%)	103 (1%)	0 (0%)
**No SBI**	2366 (88%)	7775 (97%)	0 (0%)

For temperature 0.5°C was added for axillary or tympanic temperature measurement and the highest recorded temperature from either by the parents or the physician was used. (Severely) Ill appearance was assessed by the examining physician. IQR= InterQuartile Range, SBI= Serious Bacterial Infections

Table S2: Regression coefficients of Feverkidstool		
	Original regression coefficients	Updated regression coefficients
**Pneumonia**		
**Intercept**	-17.9	-21.3
**Age** (max 1 year, in years)	1.02	0.18
**Age** (if>1 year: age in years-1)	0.01	-0.04
**Sex** (female)	0.13	-0.19
**Temperature** (°C)	0.29	0.39
**Duration of fever** (days)	0.21	0.07
**Presence of tachypnoea**	0.44	0.34
**Presence of tachycardia**	-0.04	0.32
**Oxygen saturation <94%**	1.59	1.56
**Capillary refill time ≥3s**	-0.18	0.47
**Presence of chest wall retractions**	0.47	0.65
**Ill appearance**	0.16	0.74
**Ln(CRP)** (mg/L)	0.64	0.68
**Other SBI**		
**Intercept**	-4.7	-6.2
**Age** (max 1 year, in years)	-1.73	0.32
**Age** (if>1 year: age in years-1)	0.11	0.04
**Sex** (female)	0.70	0.39
**Temperature** (°C)	-0.02	-0.01
**Duration of fever** (days)	-0.03	-0.13
**Presence of tachypnoea**	-0.11	0.33
**Presence of tachycardia**	-0.02	0.02
**Oxygen saturation <94%**	-3.29	-23.39
**Capillary refill time ≥3s**	0.30	0.85
**Presence of chest wall retractions**	-3.78	-0.61
**Ill appearance**	0.27	0.64
**Ln(CRP)** (mg/L)	1.14	0.72

Tachypnoea and tachycardia were defined according to the age-specific APLS-criteria. For temperature 0.5°C was added for axillary or tympanic temperature measurement and the highest measurement by either the parents or the physician was used. (Severely) Ill appearance was assessed by the examining physician.

2) Craig-model

Table S3: Variables of Craig-model				
**Original variable**	Proxy variable from ERNIE2	Distribution in derivation study (n=15801)	Distribution in validation study (n=8211)	Missing
Predictors				
**Age group:**	Age (years) (mean and IQR)	Not reported	1.95 (0.97 – 4.02)	0 (0%)
0-3 months	1 month – 3 months	756 (5%)	143(2%)	
>3 months - <3 years	>3 months - <3 years	11653 (74%)	5207 (63%)	
>3 years - <5 years	>3 years – 16 years	3392 (21%)	2861 (35%)	
**Male**		8825 (56%)	4401 (54%)	0 (0%)
**Duration of illness** (hours)	Duration of fever (days) (mean and IQR)	Not reported	2 (1 – 3)	1708 (21%)
**Respiratory symptoms**	Dyspnoea as reported by parents	11376 (72%)	1178 (15%)	310 (4%)
**Diarrhoea**		4031 (26%)	1514 (19%)	157 (2%)
**Urinary symptoms**	"Does your child urinate less?"	319 (2%)	932 (12%)	371 (5%)
**General appearance:**	Does the child appear severely ill?			
Mildly unwell	Not present	7874 (50%)		
Moderately unwell	Not present	1407 (9%)		
Very unwell	Yes	64 (0.4%)	702 (9%)	283 (3%)
**Breathing difficulty**	Either nasal flaring, chest wall retractions, dyspnoea observed by physician or cyanosis	2157 (14%)	759 (9%)	64 (0.8%)
**Bulging fontanelle:**				979 (12%)
Yes		42 (0.3%)	6 (0.001%)	
Closed		6462 (41%)	4709 (65%)	
**Chronic disease**		1999 (13%)	214 (3%)	Free text space
**Cough**		8515 (54%)	4785 (59%)	117 (1%)
**Chest crackles**		1314 (8%)	651 (8%)	276 (3%)
**Crying**	"Is your child weepy?"	5216 (33%)	2737 (34%)	204 (2%)
**Abnormal ENT**	Either suppurative tonsillitis, signs of acute otitis media, extensive adenopathy, redness or swelling of face or purulent conjunctivae	8571 (54%)	2398 (30%)	196 (2%)
**Felt hot**	Not present	14592 (92%)		
**Fluid intake:**				
Small decrease	Not present	6332 (40%)		
Moderate decrease	Either "Does your child eat/drink less?" or abnormal skin turgor	2088 (13%)	3688 (45%)	52 (0.6%)
None	Not present	37 (0.5%)		

**Abnormal chest sounds**	Either reduced breath sounds or ronchi	2482 (16%)	1979 (25%)	211 (3%)
**Elevated heart rate**		6847 (43%)	397 (13%)	5128 (62%)
**Elevated respiratory rate**		2214 (27%)	718 (30%)	5852 (72%)
**Meningococcal vaccine (unvaccinated)**	Not present	5347 (34%)		
**Pneumococcal vaccine (unvaccinated)**	Not present	5337 (34%)		
**Rash**	Registered in free text space 'Other signs of illness'	2778 (18%)	87 (1%)	Free text space
**Capillary refill time:**				3621 (44%)
2-3 secs		670 (4%)	1703 (37%)	
≥3 secs		48 (0.3%)	337 (7%)	
**Stridor**	Registered in free text space 'Other signs of illness'	281 (2%)	5 (0.001%)	Free text space
**Highest recorded temperature** (°C):	Highest temperature measured either by parents or physician			0 (0%)
38-<39		5634 (36%)	2010 (24%)	
39-<40		5002 (32%)	3196 (39%)	
≥40		1721 (11%)	1613 (20%)	
**Audible wheeze**	Registered in free text space 'Other signs of illness'	1018 (6%)	35 (0.4%)	Free text space
Outcome				
**Pneumonia**		533 (3.4%)	171 (2%)	0 (0%)
**Complicated urinary tract infection**		543 (3.4%	57 (1%)	0 (0%
**Bacteraemia**	Bacteraemia and sepsis	64 (0.4%)	7 (0.1%)	0 (0%)

Elevated respiratory rate and elevated heart rate were defined according to the age-specific APLS-criteria. For temperature 0.5°C was added for axillary or tympanic temperature measurement. IQR= InterQuartile Range

Table S4: Regression coefficients of Craig-model			
**Original variable**	Proxy variable from ERNIE2	Original regression coefficient	Updated regression coefficient
	**Pneumonia**		
**Intercept**		-5.10	-5.79
**Duration of illness** (hours)	Duration of fever (days)	0.002	0.0009
**Respiratory symptoms**	Dyspnoea as reported by parents	0.03	0.07
**Diarrhoea**		-0.03	0.0006
**Urinary symptoms**	"Does your child urinate less?"	0.51	0.27
**General appearance:**	Does the child appear severely ill?		
Mildly unwell	Not present	0.36	
Moderately unwell	Not present	0.71	
Very unwell	Yes	1.70	0.92
**Breathing difficulty**	Either nasal flaring, chest wall retractions, dyspnoea observed by physician or cyanosis	0.82	0.75
**Bulging fontanelle:**			
Yes		-0.13	-5.21
Closed		-0.19	-0.03
**Chronic disease**		0.68	0.43
**Age group:**			
0-3 months	1 month – 3 months	0	0
>3 months - <3 years	>3 months - <3 years	-0.60	-0.58
>3 years - <5 years	>3 years – 16 years	-0.50	-0.19
**Cough**		1.21	0.81
**Chest crackles**		0.40	0.46
**Crying**	"Is your child weepy?"	0.10	0.16
**Abnormal ENT**	Either suppurative tonsillitis, signs of acute otitis media, extensive adenopathy, redness or swelling of face or purulent conjunctivae	-0.42	-0.30
**Felt hot**	Not present	0.31	
**Fluid intake:**			
Small decrease	Not present	0.05	
Moderate decrease	Either "Does your child eat/drink less?" or abnormal skin turgor	0.04	0.16
None	Not present	-0.11	
**Male**		-0.16	0.04
**Abnormal chest sounds**	Either reduced breath sounds or ronchi	0.81	0.61
**Elevated heart rate**		0.38	0.39
**Elevated respiratory rate**		0.42	0.05

**Meningococcal vaccine (unvaccinated)**	Not present	-0.008	
**Pneumococcal vaccine (unvaccinated)**	Not present	0.38	
**Rash**	Registered in free text space 'Other signs of illness'	-0.68	-0.57
**Capillary refill time:**			
2-3 secs		0.63	0.32
≥3 secs		0.53	0.56
**Stridor**	Registered in free text space 'Other signs of illness'	-0.90	0.20
**Highest recorded temperature** (°C):	Highest temperature measured either by parents or physician		
38-<39		-0.02	0.35
39-<40		0.60	0.89
≥40		0.84	1.47
**Audible wheeze**	Registered in free text space 'Other signs of illness'	-0.96	-0.22
**Complicated urinary tract infections**			
**Intercept**		-2.96	-9.70
**Duration of illness** (hours)	Duration of illness (days)	0.0009	0.0003
**Respiratory symptoms**	Dyspnoea as reported by parents	-0.35	-0.85
**Diarrhoea**		-0.37	0.01
**Urinary symptoms**	"Does your child urinate less?"	1.66	0.44
**General appearance:**	Does the child appear severely ill?		
Mildly unwell	Not present	0.31	
Moderately unwell	Not present	0.81	
Very unwell	Yes	1.46	0.96
**Breathing difficulty**	Either nasal flaring, chest wall retractions, dyspnoea observed by physician or cyanosis	-0.08	0.10
**Bulging fontanelle:**			
Yes		-0.42	-5.59
Closed		-0.57	-0.47
**Chronic disease**		0.85	0.19
**Age group:**			
0-3 months	1 month – 3 months	0	0
>3 months - <3 years	>3 months - <3 years	-1.16	-0.93
>3 years - <5 years	>3 years – 16 years	-1.77	-1.51
**Cough**		-0.40	-0.25
**Chest crackles**		0.05	-0.40

**Crying**	"Is your child weepy?"	0.25	-0.01
**Abnormal ENT**	Either suppurative tonsillitis, signs of acute otitis media, extensive adenopathy, redness or swelling of face or purulent conjunctivae	-0.38	-0.62
**Felt hot**	Not present	0.80	
**Fluid intake:**			
Small decrease	Not present	-0.04	
Moderate decrease	Either "Does your child eat/drink less?" or abnormal skin turgor	-0.11	-0.04
None	Not present	1.24	
**Male sex**		-0.27	-0.68
**Abnormal chest sounds**	Either reduced breath sounds or ronchi	-0.31	-0.14
**Elevated heart rate**		0.21	0.15
**Elevated respiratory rate**		-0.20	0.44
**Meningococcal vaccine (unvaccinated)**	Not present	0.61	
**Pneumococcal vaccine (unvaccinated)**	Not present	-0.06	
**Rash**	Registered in free text space 'Other signs of illness'	-0.55	-5.04
**Capillary refill time:**			
2-3 secs		0.02	0.56
≥3 secs		0.52	0.99
**Stridor**	Registered in free text space 'Other signs of illness'	-1.27	-6.10
**Highest recorded temperature** (°C):	Highest temperature measured either by parents or physician		
38-<39		0.45	5.83
39-<40		0.92	6.63
≥40		0.81	6.75
**Audible wheeze**	Registered in free text space 'Other signs of illness'	-0.74	-5.21
**Bacteraemia**			
**Intercept**		-6.17	-15.05
**Duration of illness** (hours)	Duration of illness (days)	0.002	-0.007
**Respiratory symptoms**	Dyspnoea as reported by parents	-0.36	1.26
**Diarrhoea**		-0.70	1.31
**Urinary symptoms**	"Does your child urinate less?"	-0.17	-2.57

**General appearance:**	Does the child appear severely ill?		
Mildly unwell	Not present	1.04	
Moderately unwell	Not present	2.07	
Very unwell	Yes	3.42	-2.80
**Breathing difficulty**	Either nasal flaring, chest wall retractions, dyspnoea observed by physician or cyanosis	0.44	0.27
**Bulging fontanelle:**			
Yes		0.74	-6.78
Closed		0.10	1.20
**Chronic disease**		0.89	-5.90
**Age group:**			
0-3 months	1 month – 3 months	0	0
>3 months - <3 years	>3 months - <3 years	-1.64	0.60
>3 years - <5 years	>3 years – 16 years	-1.27	1.77
**Cough**		0.20	1.48
**Chest crackles**		1.14	-4.45
**Crying**	"Is your child weepy?"	0.66	-0.17
**Abnormal ENT**	Either suppurative tonsillitis, signs of acute otitis media, extensive adenopathy, redness or swelling of face or purulent conjunctivae	-1.19	0.94
**Felt hot**	Not present	0.28	
**Fluid intake:**			
Small decrease	Not present	0.08	
Moderate decrease	Either "Does your child eat/drink less?" or abnormal skin turgor	-0.51	-0.47
None	Not present	1.36	
**Male sex**		-0.16	0.50
**Abnormal chest sounds**	Either reduced breath sounds or ronchi	-1.20	0.29
**Elevated heart rate**		0.86	0.02
**Elevated respiratory rate**		-0.68	-3.90
**Meningococcal vaccine (unvaccinated)**	Not present	-0.33	
**Pneumococcal vaccine (unvaccinated)**	Not present	0.49	
**Rash**	Registered in free text space 'Other signs of illness'	0.47	-3.88
**Capillary refill time:**			
2-3 secs		0.02	0.56
≥3 secs		1.15	0.01

**Stridor**	Registered in free text space 'Other signs of illness'	-11.29	0.09
**Highest recorded temperature** (°C):	Highest temperature measured either by parents or physician		
38-<39		0.38	-8.46
39-<40		0.57	4.62
≥40		1.00	5.68
**Audible wheeze**	Registered in free text space 'Other signs of illness'	-0.87	-2.56

Elevated respiratory rate and elevated heart rate were defined according to the age-specific APLS-criteria. For temperature 0.5°C was added for axillary or tympanic temperature measurement.

3) SBI-model and SBI-score

Table S5: Variables of SBI-model and SBI-score				
**Variable**	Proxy variable in ERNIE2	Distribution in derivation study (n=1951)	Distribution in validation study (n=8049)	Missing
**Age** (years) (median and range)		1.58 (0.08 – 15)	1.96 (0.08 – 16.99)	
**Sex** (female)		850 (44%)	3734 (46%)	
Predictors				
**History of developmental delay**	Chronic disease concerning developmental delay	28 (1%)	51 (1%)	Free text space
**Risk factor for infection**	Chronic disease concerning risk factor for infection	53 (3%)	79 (1%)	Free text space
**State variation:**				37 (0.5%)
Eyes close briefly	Either “Is your child sleepy?” or drowsy appearance	31 (2%)	1638 (20%)	
Falls asleep	Either “Is your child hard to wake?” or reduced consciousness	1 (0.1%)	184 (2%)	
**Temperature category** (°C)	Highest of measured temperature by parents or physician			0 (0%)
37.5 – 38.4		205 (12%)	1153 (14%)	
≥38.4		305 (18%)	5959 (74%)	
**Capillary refill time ≥ 2s**		40 (2%)	341 (10%)	4525 (56%)
**Dehydration category**				333 (4%)
Dry mucous membranes	Either pale appearance or reduced peripheral circulation	102 (5%)	347 (4%)	
Reduced skin turgor	Either abnormal skin turgor or sunken fontanelle	9 (0.5%)	110 (1%)	
**Tachypnoea**		932 (59%)	709 (30%)	5719 (71%)
**Hypoxia category**				5412 (67%)
Mild hypoxia		265 (14%)	141 (5%)	
Severe hypoxia		66 (3%)	71 (3%)	
Outcome				
**Serious bacterial infections**		74 (3.8%)	274 (3%)	0 (0%)

Risk factor for infection = documented history of any condition known to increase a child’s risk of invasive bacterial infection. For temperature 0.5°C was added for axillary or tympanic temperature measurement. Tachypnoea was defined according to age-specific APLS-criteria. Mild hypoxia = $SaO_2 < 95\%$ and/or documented oxygen therapy, severe hypoxia = $SaO_2 < 90\%$ and/or $SaO_2 < 92\%$ despite documented oxygen therapy.

Table S6: Regression coefficients of SBI-model and points of SBI-score				
**Original variable**	Proxy variable in ERNIE2	Original regression coefficient	Updated regression coefficient	Points in risk score
**Intercept**		0	-4.78	
**History of developmental delay**	Chronic disease concerning developmental delay	1.68	0.87	4
**Risk factor for infection**	Chronic disease concerning risk factor for infection	1.44	0.90	2
**State variation:**				
Eyes close briefly	Either "Is your child sleepy?" or drowsy appearance	0.23	0.05	1
Falls asleep	Either "Is your child hard to wake?" or reduced consciousness	0.46	0.10	2
**Temperature category** (°C)	Highest of measured temperature by parents or physician			
37.5 – 38.4		0.66	0.67	1
≥38.4		1.32	1.34	2
**Capillary refill time ≥ 2s**		0.66	0.60	1
**Dehydration category**				
Dry mucous membranes	Pale appearance	1.37	0.87	2
Reduced skin turgor	Either abnormal skin turgor or sunken fontanelle or reduced peripheral circulation	2.74	1.74	4
**Tachypnoea**		0.18	0.33	1
**Hypoxia category**				
Mild hypoxia		0.47	0.52	1
Severe hypoxia		0.92	1.04	2

Risk factor for infection = documented history of any condition known to increase a child's risk of invasive bacterial infection. Tachypnoea was defined according to age-specific APLS-criteria. Mild hypoxia = SaO_2 < 95% and/or documented oxygen therapy, severe hypoxia = SaO_2 < 90% and/or SaO_2 < 92% despite documented oxygen therapy.

4) PAWS

Table S7: Variables of PAWS					
**Original variable**	Proxy variable in ERNIE2	Distribution in internal validation study		Distribution in external validation study (n=8211)	Missing
		Cases (n=46)	*Controls (n=49)*		
**Age** (mean and range)		5.5 (0 - 16)	5.9 (0 - 16)	3.16 (0.08 – 16.99)	
**Sex (female)**		20 (43%)	25 (51%)	3810 (46%)	
Predictors					
**Respiratory rate** (breaths per minute) (median and IQR)		Not reported		30 (20 – 40)	5852 (71%)
**Work of breathing**					
Mild intercostal recession	Chest wall retractions			150 (2%)	171 (2%)
Severe intercostal recession, tracheal tug	Not present				
**SaO_2** (%) (median and IQR)				97 (97 – 98)	5543 (68%)
**Temperature** (°C) (median and IQR)	Highest of measured temperature by parents or physician			39 (38.4 – 39.7)	0 (0%)
**Capillary refill** (s) (median and IQR)				2 (1 – 2)	4590 (56%)
**Heart rate** (median and IQR)				110 (92 – 130)	5128 (62%)
**AVPU scale**					43 (1%)
Alert				7718 (94%)	
Responds to voice	Either "Is your child hard to wake?" or drowsy appearance			420 (5%)	
Responds to pain	Reduced consciousness			30 (0.4%)	
Unresponsive	Not present				
Outcome					
**PAWS-score** (median and IQR)		5 (NA)	0 (NA)	2 (1 – 2)	0 (0%)
**Admission to PICU**	Admission to PICU or ED referral	46 (100%)	49 (0%)	47 (1%)	2228 (27%)
	Serious infections	Not reported		498 (6%)	0 (0%)

For temperature 0.5°C was added for axillary or tympanic temperature measurement. IQR= InterQuartile Range, NA = not reported

3) Sensitivity analyses

Supplementary table 8: Discriminative ability of feverkidstool: sensitivity analysis

	Pneumonia	Other SBI
**Full dataset** (n=8047)	0.80 (0.77 – 0.84)	0.74 (0.70 – 0.79)
**Emergency department** (n=2450)	0.78 (0.72 – 0.83)	0.67 (0.61 – 0.73)
**CRP-result available** (n=6425)	0.79 (0.75 – 0.83)	0.71 (0.66 – 0.76)
**Complete vital parameters** (n=989)	0.65 (0.53 – 0.72)	0.63 (0.46 – 0.80)

SBI = Serious Bacterial Infections

Supplementary table 9: Discriminative ability of Craig-model: sensitivity analysis

	Pneumonia	Complicated UTI	Bacteremia
**Setting**	C-statistic (95%-CI)	C-statistic (95%-CI)	C-statistic (95%-CI)
**Entire study population** (n= 8211)	0.80 (0.77 – 0.83)	0.75 (0.70 – 0.80)	0.63 (0.39 – 0.88)
**Age 1 month to 5 years** (n= 6993)	0.78 (0.75 – 0.82)	0.74 (0.69 – 0.80)	0.64 (0.40 – 0.87)
**Emergency department** (n= 2590)	0.76 (0.71 – 0.80)	0.64 (0.57 – 0.72)	0.68 (0.47 – 0.88)

UTI = Urinary Tract Infection, CI = Confidence Interval

Supplementary table 10: Discriminative ability of SBI-model: sensitivity analysis

	C-statistic (95%-CI)
**Full dataset** (n=8047)	0.66 (0.59 – 0.73)
**Emergency department** (n=2450)	0.64 (0.56 – 0.70)
**Complete vital parameters** (n=1063)	0.69 (0.58 – 0.77)

SBI = Serious Bacterial Infections, CI = Confidence Interval

Supplementary table 11: Diagnostic test parameters of PAWS: sensitivity analysis

	Serious infections (n= 8211)	Emergency department or PICU referral (n= 8211)	Emergency department (n= 2590)	Complete vital parameters (n=1001)
Parameter	Value (95%-CI)	Value (95%-CI)	Value (95%-CI)	Value (95%-CI)
Sensitivity	0.32 (0.28 - 0.37)	0.40 (0.26 - 0.56)	0.29 (0.24 - 0.34)	0.72 (0.60 - 0.83)
Specificity	0.86 (0.85 - 0.87)	0.85 (0.84 - 0.86)	0.86 (0.84 - 0.87)	0.65 (0.62 - 0.68)
LR(+)	2.28 (1.98 - 2.61)	2.67 (1.88 - 3.79)	2.04 (1.69 - 2.47)	2.08 (1.76 – 2.47)
LR(-)	0.79 (0.74 - 0.84)	0.70 (0.55 - 0.89)	0.83 (0.77 - 0.89)	0.42 (0,29 – 0.62)

PICU = Paediatric Intensive Care Unit, CI = Confidence Interval, LR(+) = Positive Likelihood Ratio, LR(-) = Negative Likelihood Ratio
